# Evaluation in Nonhuman Primates of Vaccines against Ebola Virus

**DOI:** 10.3201/eid0805.010284

**Published:** 2002-05

**Authors:** Thomas W. Geisbert, Peter Pushko, Kevin Anderson, Jonathan Smith, Kelly J. Davis, Peter B. Jahrling

**Affiliations:** * U.S. Army Medical Research Institute of Infectious Diseases, Fort Detrick, Maryland, USA

**Keywords:** Keywords: Ebola, macaque, vaccine, Vaccinia virus, replicon

## Abstract

Ebola virus (EBOV) causes acute hemorrhagic fever that is fatal in up to 90% of cases in both humans and nonhuman primates. No vaccines or treatments are available for human use. We evaluated the effects in nonhuman primates of vaccine strategies that had protected mice or guinea pigs from lethal EBOV infection. The following immunogens were used: RNA replicon particles derived from an attenuated strain of *Venezuelan equine encephalitis virus* (VEEV) expressing EBOV glycoprotein and nucleoprotein; recombinant *Vaccinia virus* expressing EBOV glycoprotein; liposomes containing lipid A and inactivated EBOV; and a concentrated, inactivated whole-virion preparation. None of these strategies successfully protected nonhuman primates from robust challenge with EBOV. The disease observed in primates differed from that in rodents, suggesting that rodent models of EBOV may not predict the efficacy of candidate vaccines in primates and that protection of primates may require different mechanisms.

Ebola virus (EBOV) and *Marburg virus* (MBGV), which make up the family *Filoviridae*, cause severe hemorrhagic disease in humans and nonhuman primates, killing up to 90% of those infected. EBOV was first recognized in the former Zaire in 1976. Subsequently, outbreaks have been documented in Sudan, Gabon, the former Zaire, Côte d’Ivoire, and Uganda (1-3). In addition to the African outbreaks, the species *Reston Ebola virus*, which may be less pathogenic for humans, was isolated from cynomolgus monkeys imported from the Philippines to the United States (4). Although outbreaks of EBOV have been self-limiting, the lack of an effective vaccine or therapy has raised public health concerns about these emerging pathogens.

In early attempts to develop a vaccine against EBOV, guinea pigs or nonhuman primates were vaccinated with formalin-fixed or heat-inactivated virion preparations. Results from these studies were inconsistent: Lupton et al. (5) partially protected guinea pigs against EBOV, while Mikhailov et al. (6) achieved complete protection of four of five hamadryad baboons by vaccinating them with an inactivated EBOV vaccine. However, other studies suggested that inactivated EBOV did not induce sufficient immunity to reliably protect hamadryl baboons against a lethal challenge (7). Conventional strategies of attenuating viruses for use as human vaccines have not been pursued for EBOV because of concerns about reversion to a wild-type form. However, the possibility of following this strategy by using newly developed infectious clones of EBOV may now be feasible (8).

Recent efforts have focused on the use of recombinant DNA techniques to stimulate cytotoxic T-lymphocyte responses. Vaccinating guinea pigs with plasmids against EBOV nucleoprotein (NP), soluble glycoprotein, or glycoprotein (GP) elicited humoral and cellular immune responses against these gene products but only partially protected them against lethal challenge (9). However, results of this study were difficult to interpret because all the guinea pigs were killed 10 days after EBOV challenge, which is within the expected survival time for untreated animals (8-14 days) (10). In 2000, Sullivan et al. (11) reported protection of cynomolgus monkeys from EBOV infection by injecting them with naked-DNA GP, followed by an adenovirus-expressing GP booster. Results of this study document the feasibility of vaccination against EBOV. However, these results require confirmation and further evaluation, as a low dose (6 PFU) was used for the challenge. Other studies reported a protective effect of EBOV vaccination with a low infective challenge dose (10 50% lethal doses [LD_50_]) (7); however, all vaccinated animals in these dosing studies died after receiving higher infective doses (100 and 1,000 LD_50_), which may more accurately mimic natural or nosocomial exposures.

Our efforts to develop a vaccine against EBOV focused on several potential vaccine candidates. First, we used *Venezuelan equine encephalitis virus* (VEEV) replicon particles (VRP) expressing EBOV genes known to protect guinea pigs and mice from EBOV disease (10); VRP expressing MBGV genes also protected guinea pigs and cynomolgus monkeys against MBGV (12). Second, we used a recombinant *Vaccinia virus* (VACV) system expressing EBOV GP and demonstrated that this vector protected guinea pigs from EBOV hemorrhagic fever (13). A third strategy used encapsulated, gamma-irradiated EBOV particles in liposomes containing lipid A (14); and the fourth approach evaluated vaccination with a concentrated, gamma-irradiated whole-virion preparation. None of these approaches, which successfully protected rodents from lethal infection, were protective for cynomolgus or rhesus macaques challenged with EBOV.

## Materials and Methods

Cynomolgus macaques (*Macaca fascicularis*) or rhesus macaques (*M. mulatta*) weighing 4 to 6 kg were used. For vaccine studies with VEE replicons, EBOV GP or NP genes were introduced into the VEEV RNA as described (10). Groups of three cynomolgus macaques were vaccinated with VRP that expressed EBOV GP, EBOV NP, a mixture of EBOV GP and EBOV NP, or a control antigen (influenza hemagglutinin) that has no effect on EBOV immunity. Animals were vaccinated by subcutaneous injection of 10^7^ focus-forming units of VRP in a total of 0.5 mL at one site. Vaccinations were repeated 28 days after the first injection and 28 days after the second.

In conducting research with animals, the investigators followed the Guide for the Care and Use of Laboratory Animals prepared by the Committee on Care and Use of Laboratory Animals of the Institute of Laboratory Animal Resources, National Research Council (1996). The animal facilities and animal care and use program of the U.S. Army Medical Research Institute of Infectious Diseases are accredited by the Association for Assessment and Accreditation of Laboratory Animal Care International.

For vaccine studies using primates, we adapted the optimal immunization regimens determined from the rodent studies. For the vaccine based on recombinant VACV, the EBOV GP gene was inserted into a VACV transfer vector plasmid, and recombinant VACV expressing EBOV GP were isolated as reported (13). Three cynomolgus macaques were injected subcutaneously with the EBOV GP-expressing VACV vector. Injections were repeated at 28 and 53 days after the first injection.

For vaccine studies with inactivated EBOV whole-virion preparation, viral particles were concentrated from Vero cell culture fluids by ultracentrifugation in a sucrose density gradient. The infectivity titer of the preparation was approximately 8.0 log_10_ PFU/mL. The preparation was inactivated by exposure to ^60^Co gamma rays (6 x 10^6^ rads). The absence of residual infectivity was proven by exhaustive testing for residual infectivity in assays in Vero cells (15,16). Two cynomolgus monkeys and two rhesus monkeys were injected subcutaneously with a 50-µg dose of the gamma-irradiated virion preparation in RIBI adjuvant (Corixa, Hamilton, MT). As a further check on complete viral inactivation, blood samples taken from the monkeys 3 and 5 days after they received the vaccine were free of infectious viremia. Injections were repeated at days 7 and 35 after the initial injection.

For vaccine studies using a liposome formulation, three cynomolgus monkeys were vaccinated with gamma-irradiated virus encapsulated in liposomes containing lipid A, as described for previous studies in mice (14). Animals received 1.0 mL of the liposome preparation by intravenous injections that were repeated at 28 and 55 days after the initial vaccination. Four macaques (two cynomolgus and two rhesus) served as unvaccinated controls for the VACV, gamma-inactivated virion, and liposome studies.

Anti-EBOV neutralizing antibody titers were monitored by measuring plaque reduction in a constant virus:serum dilution format (15). All macaques received intramuscular injections in the leg with 1,000 PFU of the Zaire subtype of EBOV, which was isolated from a human patient in 1995 (16). Blood was obtained from all monkeys under Telazol anesthesia (Fort Dodge Laboratories, Fort Dodge, IA) at 2- or 3-day intervals postinfection to determine infectious viremia, neutralizing antibody titers, and standard hematologic and clinical pathology parameters. All terminally ill monkeys were killed and necropsied for pathologic examination. Virus infectivity assays on plasma and tissue homogenates were done by forming plaques on Vero cell monolayers as described (15,16).

Tissues were immersion fixed in 10% neutral-buffered formalin and processed for histopathologic and immunohistochemical characteristics as described (17-19). Replicate sections of spleen were stained with phosphotungstic acid hematoxylin to demonstrate polymerized fibrin. Sections of spleen from five EBOV-infected guinea pigs and five mice from previous studies (20,21) were similarly stained for polymerized fibrin. Portions of selected tissues from 11 monkeys were also immersion fixed in 4% formaldehyde and 1% glutaraldehyde and processed for transmission electron microscopy according to conventional procedures (17-19).

## Results

### Serologic Response

Prechallenge EBOV neutralization titers were measured for the 26 nonhuman primates used in this study (Table 1). Although all vaccinated animals seroconverted by immunoglobulin G enzyme-linked immunosorbent assay, neutralizing antibody (PRNT_50_) titers were very low. Only one macaque vaccinated with VRP-expressed EBOV GP had detectable neutralizing antibody. The marginal PRNT did not preclude challenge of the monkeys; however, in previous studies, similar results were obtained when cynomolgus macaques were vaccinated with the VRP expressing MBGV genes, yet the animals were protected from lethal disease (12).

**Table 1:** Prechallenge neutralization titers of Ebola virus (EBOV)-vaccinated monkeys

Nonhuman primate species	No. of animals	Vector	Antigen	Neutralization titers^a^
Cynomolgus	3	Replicon	GP	0, 0, 0
Cynomolgus	3	Replicon	NP	0, 0, 0
Cynomolgus	3	Replicon	GP + NP	0, 0, 10
Cynomolgus	3	Replicon	Influenza HA	0, 0, 0
Cynomolgus	3	Vaccinia	GP	10, 20, 20
Cynomolgus	3	Liposome	Inactivated virion	20, 40, 80
Cynomolgus	2		Inactivated virion	10, 20
Rhesus	2		Inactivated virion	10^b^, 20
Cynomolgus	2	None		0, 0
Rhesus	2	None		0, 0

^a^Immunoglobulin G enzyme-linked immunosorbent assay, neutralizing antibody (PRNT_50_) All vaccinated monkeys seroconverted by enzyme-linked immunosorbent assay before challenge. ^b^Animal survived challenge. GP, glycoprotein; NP, nucleoprotein.

### Challenge of Vaccinated Monkeys with EBOV

All animals, including the four untreated macaques, were challenged with 1,000 PFU of EBOV. Timing of challenge varied because of differences in the optimal immunization regimens determined by preliminary testing in rodents. VRP-vaccinated animals were challenged 49 days after the third vaccine dose. At postchallenge day 3, all animals became ill; two animals from each vaccination group (i.e., GP, NP, GP + NP, influenza HA) died on day 6, and the remaining animals died on day 7 (Table 2). VACV GP-inoculated macaques were challenged 45 days after the third vaccine dose, EBOV liposome-vaccinated animals 35 days after the third vaccine dose, and macaques vaccinated with the gamma-irradiated whole-virion preparation 43 days after the third vaccine dose. Again, all animals except one rhesus macaque, which received the gamma-irradiated virion preparation, became ill on the third day after challenge. Two cynomolgus macaques vaccinated with the gamma-irradiated virion preparation, one VACV-GP animal, and one untreated cynomolgus macaque died on postchallenge day 6 (Table 2). The two remaining VACV-GP animals died at day 7 after challenge, as did two of the animals vaccinated with the EBOV liposome preparation and the remaining untreated cynomolgus macaque. The untreated rhesus macaques died on days 8 and 9 postchallenge; one rhesus vaccinated with the gamma-irradiated virion preparation died on day 9, and the other survived challenge. The remaining animal vaccinated with the EBOV liposome preparation died 11 days after challenge. The rhesus macaque that survived challenge did not become ill during the study and had a PRNT_50_ values >320 at day 26 postchallenge and 80 at days 26, 61, 99, and 902 postchallenge.

**Table 2:** Challenge of vaccinated monkeys with Ebola virus (EBOV)

NHP Species	Vector	Antigen	Survival/total	Viremic/total	Day of death^a^
Cynomolgus	Replicon	GP	0/3	3/3	6, 6, 7
Cynomolgus	Replicon	NP	0/3	3/3	6, 6, 7
Cynomolgus	Replicon	GP + NP	0/3	3/3	6, 6, 7
Cynomolgus	Replicon	Influenza HA	0/3	3/3	6, 6, 7
Cynomolgus	Vaccinia	GP	0/3	3/3	6, 7, 7
Cynomolgus	Liposome	Inactivated virion	0/3	3/3	7, 7, 11
Cynomolgus		Inactivated virion	0/2	2/2	6, 6
Rhesus		Inactivated virion	1/2	2/2	9
Cynomolgus	None		0/2	2/2	6, 7
Rhesus	None		0/2	2/2	8, 9

^a^Number of days after challenge with 1,000 PFU of EBOV. NHP, nonhuman primate; GP, glycoprotein; NP, nucleoprotein.

### Histopathologic Examination

Conventional histopathologic and electron microscopic examination of lymphatic tissues, liver, and gastrointestinal tract showed no differences in lesions between the vaccinated animals and the unvaccinated EBOV-infected controls. Depletion and necrosis or apoptosis were noted in all lymphoid germinal centers in spleen, peripheral, and mesenteric lymph nodes, as described in other studies (17-19). The spleen had copious deposits of fibrin throughout the red pulp, as well as abundant karyorrhectic cellular debris. By electron microscopy, widespread bystander lymphocyte apoptosis was a prominent feature in all the lymphatic tissues examined. Fibrin and fibrinocellular thrombi were also prominent in the submucosa of the gastrointestinal tract and in hepatic sinusoids, again consistent with well-documented findings (17,18).

We also evaluated retrospectively EBOV-infected rodent tissues in parallel. Although sites of infection and morphologic changes between guinea pigs, mice, and nonhuman primates had many similarities, the lack of fibrin thrombi in spleen and visceral vasculature was particularly striking in the EBOV-infected mice (Figure). Fibrin deposition was seen in guinea pigs as reported (20), but fibrin deposits and thrombi were considerably less prevalent compared with deposits in nonhuman primates (Figure). Lymphocyte apoptosis was also less frequently observed by electron microscopy in rodent lymphatic tissues than in nonhuman primates. EBOV was demonstrated in liver, spleen, kidney, lung, adrenal gland, and lymph nodes of all necropsied monkeys by immunohistochemistry, electron microscopy, or virus infectivity titration.

**Figure:**
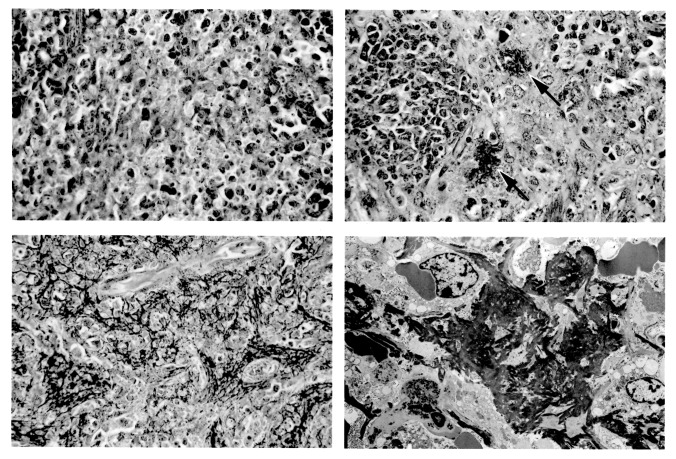
Sections of spleen from Ebola virus (EBOV)-infected animals. Top left, BALB/c mouse, note absence of polymerized fibrin (phosphotungstic acid [PTA] hematoxylin, original magnification X400). Field representative of five of five mice tested. Top right: guinea pig. Note discreet foci of polymerized fibrin (arrows) (PTA hematoxylin, original magnification X400). This field shows infrequent fibrin deposits; most fields in five of five animals examined showed no evidence of polymerized fibrin. Lower left: cynomolgus monkey. Note deposition of polymerized fibrin in red pulp (PTA hematoxylin, original magnification X400). Field representative of 25 of 25 monkeys. Lower right: cynomolgus monkey. Electron micrograph showing abundant fibrin deposits in red pulp (original magnification X5,300). Field representative of 11 of 11 monkeys examined.

## Discussion

Our results indicate that rodent models of EBOV hemorrhagic fever do not consistently predict efficacy of candidate vaccines in nonhuman primates, perhaps because the disease course in rodents differs from that reported in human and nonhuman primates (17-19,22,23). Mice do not have the hallmark disseminated intravascular coagulation (DIC) found in end-stage lesions of humans and nonhuman primates. Viremia and widespread tissue dissemination are much more apparent in nonhuman primates than in guinea pigs (20). In addition, guinea pigs have less DIC than do nonhuman primates. Lymphocyte apoptosis was not reported to be a prominent feature of EBOV infection in mice or guinea pigs (20,21) but was a consistent feature of disease in humans (24) and nonhuman primates (19). Clinical disease and related pathologic features in nonhuman primates infected with EBOV appear to more closely resemble those described in human EBOV hemorrhagic fever (22,23). Other studies have shown inconsistencies between rodent and nonhuman primate models of human hemorrhagic disease in the protective efficacy of candidate vaccines. For example, guinea pigs were protected from *Lassa virus* by VACV recombinants expressing the viral nucleoprotein (25,26); however, this vaccination strategy failed to protect rhesus macaques (27).

The effort to develop an EBOV vaccine began after the initial identification of EBOV in 1976, but 25 years later the goal remains elusive. Attempts to develop killed-virus vaccines against EBOV hemorrhagic fever have had inconsistent results (5-7). Recent progress in genetic vaccination strategies has demonstrated that immunity can be achieved against a low dose of EBOV. While protection against any lethal challenge dose of EBOV is a remarkable achievement, we have set the bar somewhat higher than 6 PFU, since a laboratory exposure through a needlestick and infected blood would likely entail a dose of at least 1,000 PFU. Therefore, our priority is to empirically develop a vaccine that protects against at least 1,000 PFU rather than to initiate an exhaustive investigation of protective immune mechanisms. We were encouraged by the demonstrated success of the VEEV replicon vector expressing MBGV glycoprotein in protecting cynomolgus macaques from challenge with homologous MBGV (12). No MBGV-neutralizing activity was observed at >1:20 dilutions in prechallenge sera of any of the MBGV GP VRP-vaccinated macaques (12), yet these animals did not become viremic, showed no signs of disease, and survived challenge. Historically, *Filovirus*-neutralizing antibodies have been difficult to demonstrate in vitro (15); while the presence of neutralizing antibodies is desirable, it is neither sufficient nor necessary to clear viral infection (16). Unfortunately, the VEEV replicon strategy that was successfully employed for MBGV in cynomolgus macaques and for EBOV in mice and guinea pigs (10) did not protect cynomolgus macaques from EBOV disease. These differences observed between EBOV and MBGV may result from differences in the course of infection. Specifically, the mean day of death for untreated cynomolgus monkeys experimentally infected intramuscularly with 1,000 PFU of EBOV (Zaire subtype) is 6.3 (n=15; data not shown), while the mean day of death for cynomolgus monkeys infected intramuscularly with a comparable dose of MBGV (Musoke isolate) is 9.1 (n=8; data not shown). Thus, macaques infected with MGBV have nearly three more days to mount an effective immune response against the challenge virus than macaques infected with EBOV (Zaire). Clearly, other variables, including differences observed between EBOV (Zaire) and MBGV with respect to GP gene expression (28), tropism, and host cell responses, may contribute to differences in disease pathogenesis and outcome of infections.

The induction of humoral and cytotoxic T-lymphocyte responses to EBOV NP and GP has been demonstrated in guinea pigs, although the relative contributions of these responses to immune protection are unclear (9). Moreover, transfer of EBOV immune serum in rodent and nonhuman primate models provided inconsistent results. Passive transfer of immune serum from VRP-vaccinated animals did not protect guinea pigs or mice against lethal challenge (10); however, transfer of hyperimmune equine immune globulin (which had high EBOV neutralization titers) to guinea pigs protected them against disease (16,29). Passive treatment of cynomolgus monkeys with the equine immune globulin delayed death but did not ultimately protect the monkeys against lethal EBOV hemorrhagic fever (16,29). In contrast, hamadryl baboons were protected against lethal EBOV challenge by passive treatment with the equine immune globulin and the use of a lower challenge dose (30). These results suggest that cell-mediated effector mechanisms may play a more important role in protection than do humoral responses. Nonetheless, the role of humoral immunity is in fact supported by studies showing consistent delay in death or protection of primates therapeutically treated with EBOV-neutralizing antibodies (16,29,30).

We conclude that, although rodent models are useful as preliminary screens for candidate vaccines and therapeutic treatments, nonhuman primates likely provide a more useful and definitive model for EBOV hemorrhagic fever in humans. Furthermore, differences in disease pathology between rodent and nonhuman primate models of EBOV suggest that protection of primates may require different protective mechanisms.
